# Incidence and outcomes of SARS-CoV-2-associated PIMS in Germany: a nationwide analysis

**DOI:** 10.1007/s15010-022-01877-w

**Published:** 2022-06-30

**Authors:** Christian Karagiannidis, Leif-Erik Sander, Marcus A. Mall, Reinhard Busse

**Affiliations:** 1 Department of Pneumology and Critical Care Medicine, Cologne-Merheim Hospital, ARDS and ECMO Center, Kliniken Der Stadt Köln, Witten/Herdecke University Hospital, Ostmerheimer Strasse 200, 51109 Cologne, Germany; 2 Department of Infectious Diseases and Respiratory Medicine, Charité-Universitaetsmedizin Berlin, corporate member of Freie Universität Berlin and Humboldt-Universität Zu, Berlin, Germany; 3 Department of Pediatric Respiratory Medicine, Immunology and Critical Care Medicine, Charité-Universitaetsmedizin Berlin, corporate member of Freie Universität Berlin and Humboldt-Universität Zu, Berlin, Germany; 4 Department of Health Care Management, Technische Universität Berlin, Berlin, Germany

Pediatric inflammatory multisystem syndrome (PIMS) is a COVID-19-linked severe inflammatory condition, which occurs in children a few weeks after SARS-CoV-2 infection [1]. Clinical presentations of PIMS often involve fever, diarrhea or shock and may lead to the development of multi-organ failure in some cases. The WHO definition further includes rash or bilateral non-purulent conjunctivitis or muco-cutaneous inflammation signs, features of myocardial dysfunction, evidence of coagulopathy, elevated markers of inflammation, and the absence of obvious other causes of inflammation. Steroids and intravenous immunoglobulins (IVIGs) are established as first-line therapy for PIMS. Since the beginning of the pandemic, many cases of PIMS have been reported worldwide, but detailed data reporting the total nationwide incidence in relation to SARS-CoV-2 infections are limited to the first months of the pandemic [2]. Most publications report case-series or smaller studies of PIMS cases and their characteristics, in addition to several published meta-analyses [3].

Here, we report nationwide, complete unselected data of PIMS incidence and outcomes for Germany, based on records from the federal German Institute for the Hospital Remuneration System (InEK) of all children (age < 18 years) hospitalized with acute COVID-19 or PIMS, discharged from hospital between 1st of January and 31st of December 2021. During this time, 1,364,857 SARS-CoV-2 infections were reported for children in Germany by the Robert-Koch-Institute (Table 1). Our analysis indicates a hospitalization rate of acute SARS-CoV-2 infections in children of 0.74%. The mean age of hospitalized pediatric COVID-19 patients was 6.2 years, and in-hospital mortality was 0.3% for pediatric COVID-19. Three percent of all hospitalized children were treated on the ICU. Children requiring ICU treatment were considerably older (mean age 13.9 years), with a mean duration-of-stay of around 13.7 days.

**Table 1:** SARS-CoV-2 positive cases in 2021 in Germany in persons aged younger than 18 years (ICD code U07.1) and cases of pediatric inflammatory multisystem syndrome (PIMS, ICD code U10.9)

	Entire population < 18 years (ICD code U07.1)		PIMS cases < 18 years (ICD code U10.9)		
		%		%	% of all SARS-CoV-2 infections
All SARS-CoV-2 Infections					
# cases	1,364,857	100%	–	–	–
Hospitalizations due to COVID-19					
# cases	10 090	0.74% of infected	1006	–	0.07%/1:1357
Mean age	6.2 years	–	7.4 years	–	–
Sex (male)	52%	–	62%	–	–
Duration of hospitalization	4.3 days	–	8.8 days	–	–
# with IvIg administration (> 2.5 gr)	–	–	683		–
Deaths	29	0.3% of hospitalized/ 1:47,064 of infected	3	0.3% of hospitalized with PIMS	1:454,952
ICU treatment in patients with COVID-19*					
# cases	351	3.0% of hospitalized/0.02% of infected	228	22.7% of hospitalized with PIMS	0.02%/1:5986
Mean age	13.9 years	–	7.6 years	-	–
# with mechanical ventilation	116	33% of those on ICU	113	50% of those on ICU	0.01%/1:12,078
# with ECMO	7	2.0% of those on ICU	7	3.1% of those on ICU	–
Duration of hospitalization	13.7 days	–	13.5 days	–	–
Of which: duration on ICU	8.1 days	–	8.8 days	–	–
Deaths	19	5.4% of those on ICU	2	0.9% of those on ICU	–

^*^ICU definition was restricted to cases for which SAPS scores were documented

In 2021, 1006 cases of PIMS were treated in hospitals in Germany. The incidence based on all reported SARS-CoV-2 infections in the same year was 0.07% (1:1357). The mean age was 7.4 years, the mean length of hospital stay was 8.8 days, and IVIG was administered in 68% of all PIMS cases, and three deaths were recorded for children hospitalized with PIMS in 2021 (0.3%). 23% of all PIMS cases in Germany were treated on the ICU, 113 (11%) of which received mechanical ventilation and seven children (0.7%) required extracorporeal membrane oxygenation. The mean age of PIMS cases treated on ICU was younger compared to children on ICU due to acute COVID-19 (7.6 vs. 13.9 years), whereas the duration of ICU stay was comparable for PIMS and acute COVID-19 (8.8 vs 8.1 days, Table. 1).

The mean length of hospitalization of 8.8 days, the requirement of ICU treatment, mechanical ventilation and IVIG application reflect a high burden of disease in COVID-19-associated PIMS. Our study has limitations. Administrative data, as those used in our study, depend on ICD coding, which might be handled slightly differently among hospitals, i.e., whether it is done directly by the treating physicians or by coding staff. The data are limited with regard to comorbidities and concomitant diseases, and the overall incidence of SARS-CoV-2 infections in children might be underestimated, even though Germany had intense regular testing strategies implemented in schools and day care facilities. We here report an incidence of PIMS cases (0.07%, 1:1357) in 2021, which is considerably higher than previous reports for the US (approximately 1: 3000) [2] and stresses the importance of unbiased and unselected health-care data as a valuable source for determining the incidence of rare conditions, despite that the granularity of these data lacks some important information. Our data are in line with more recent and nationwide data from the USA. The CDC recently reported 6851 PIMS cases until Jan. 31, 2022 [4]. Given the more than 10.8 million SARS-CoV-2 infections among children, this translates into an incidence of approximately 1:1400. Of note, vaccination is highly effective against severe COVID-19 and it was recently reported to prevent PIMS [5]. Interestingly, the incidence of PIMS is by far higher than reported for the Kawasaki Syndrome with 7:100,000, which shares many similarities with PIMS (6). Moreover, these incidences should be regarded in conjunction with a more granular database of the German Society of Pediatric Infectious Diseases of hospitalized children with proven SARS-CoV-2 infection and PIMS since March 2020. Roughly two-thirds of all pediatric departments in Germany participate in this voluntary survey, and as of 8th of May 2022, 5,532 patients have been reported, 187 of them (3.4%) requiring intensive care, most of them (72%) with severe underlying diseases. Interestingly, 81% of all hospitalized children did not receive any supportive or specific treatment for their SARS-CoV-2 infection. Since the beginning of the survey, 21 fatalities have been reported, the vast majority patients with severe underlying diseases, and more than half of them may have died for other reasons than COVID-19 (personal communication). Compared to this data, reporting in the DGPI registry focuses on more granular data, typically done directly by treating physicians, including cases where clinical data lead to the diagnosis, but possibly leading to overdiagnosis as suspicious cases are coded but not removed if diagnosis is not confirmed. This is the case with administrative data, as they depend on the final diagnosis—but may have lower granularity, but include all cases treated in German hospitals.

Thus, PIMS represents are rare, but serious complication of pediatric SARS-CoV-2 infection and the incidence is higher than previously reported, and may be reduced by vaccination.
